# Continuous Work Support Checklist for Female Healthcare Workers: Scale Development and Validation

**DOI:** 10.3390/ijerph17165875

**Published:** 2020-08-13

**Authors:** Mariko Ono, Toru Nagasawa, Takayoshi Ohkubo, Akiko Tsuchiya, Haruko Hiraike, Hiroko Okinaga, Kyoko Nomura

**Affiliations:** 1 Department of Medicine, Teikyo University School of Medicine, Tokyo 173-8605, Japan; malinkopf@gmail.com (M.O.); wgdn1954@yahoo.co.jp (T.N.); 2 Department of Hygiene and Public Health, Teikyo University School of Medicine, Tokyo 173-8605, Japan; tohkubo@med.teikyo-u.ac.jp; 3 Department of Nursing, Teikyo University Hospital, Tokyo 173-8605, Japan; a-tutiya@med.teikyo-u.ac.jp; 4 Department of Obstetrics and Gynecology, Teikyo University School of Medicine, Tokyo 173-8605, Japan; haruko.hiraike@gmail.com; 5 Support Center for Women Physicians and Researchers, Teikyo University, Tokyo 173-8605, Japan; hiokinaga@gmail.com; 6 Department of Environmental Health Science and Public Health, Akita University Graduate School of Medicine, Akita 010-0825, Japan

**Keywords:** women, continuous work, healthcare workers, intention to leave, scale development, workplace, checklist

## Abstract

Healthcare jobs are very popular among women, however in Japan, women readily quit working because of gender-role responsibilities. This study aimed to develop a workplace support checklist for women to continue to work. In 2017, we investigated 780 (female 74.8%) faculty members and healthcare professionals of one medical university in Japan. We asked them to score the extent to which they considered 35 items identified by a task team, to be related to continuous work support for female workers in healthcare. We carried out an exploratory factor analysis and extracted four domains with 16 items in all: “Support for child rearing and home care” (five items), “Information dissemination” (five items), “Active promotion of women workers to higher positions” (three items), and “Consulting and counseling service” (three items), with Cronbach’s alpha values ranging from 0.88 to 0.92. We found that the first three factors were generally associated with reasonably relevant characteristics of being female, in their 30s, married, and members of faculty. We also found that women with “Intention to leave” the workplace underscored the importance of “Support for child rearing and home care” and “Consulting and counseling service”. These results suggest that the checklist is reliable and valid.

## 1. Introduction

According to the recent labor force survey in Japan, the number of working women exceeds 29 million, but 56% of them are part-time workers [1]. Why do Japanese women not work as long as their male counterparts? The Gender and Diversity White Paper revealed that nearly 50% of women quit work after their first child’s birth [2]. The National Gender Opinion Survey found that 43% of women agreed with the gender division of labor, that men should be the breadwinners and women should shoulder family responsibilities [3]. Hence, the concept of stereotypical gender roles is deeply embedded in the Japanese mindset, including in women themselves [4]. In Japan, once women quit working, they are more likely to drop out of the promotion race because the Japanese employment system is based on full-time labor and life-time employment. Even if women shifted from full-time to part-time work, they would instantly lose the opportunity for promotion to higher positions, proper employee contracts, and salary raises. In short, the Japanese industry continues to prefer a long working engagement [5]. This Japanese working style further influences the gender gap with regard to parental leave; only 3% of men take parental leave, while more than 80% of women do so in Japan, exhibiting a strong gender division of labor. The small number of women candidates in decision-making positions in enterprises in Japan further explains why Japan has a large gender gap index and is ranked 110th among 149 nations [6].

Such a circumstance, which prevents women from continuing to work, exists in the medical and health-related fields as well. It should be noted that, ironically, these areas have the largest numbers of women workers in Japan [7]. Historically, medicine is a male-dominated professional field that does not include women-friendly workplaces at times. For example, medical institutions and patients appreciate long working hours which compensates for the shortage of physicians [8]. However, under such circumstance, women have no choice but to sacrifice their gendered role and family responsibilities to pursue their career the way that men do. Consequently, in Japan, when women’s career conflicts with their personal responsibilities—such as child-rearing or elderly-care—the majority quits full-time labor [9]. Subsequently, women in medicine are less likely to obtain promotion opportunities [10], high income [11], and research funding [12]. Our previous report demonstrated that, even among surgeons, men earn more than women adjusting for subspecialty [13]. Therefore, gender inequality at workplace hampers work motivation for women. This is supported by our previous study of eight medical schools in Japan, which concluded that women who perceived gender inequality at workplace were more likely to stop working full-time [14]. A study in Canada [15] previously highlighted the importance of the organizational climate for gender equity and demonstrated that the gender gap in academic medicine may have negative consequences for organizational effectiveness and workplace culture. We also found that utilizing women and absence of inequality or harassment at workplace alleviated women’s burnout [16]. These findings suggest that organizational fairness that improves gender equity may be a useful tool to enable women to continue working. In addition to childcare facilities at the workplace, workplace support is especially important for female health workers to continue working. These may include information related to parental and home-care leave, flexible working conditions, or the active employment and promotion of women [17,18,19]. In a previous study, we reported the importance of including consultation services at the workplace to help with career, work, harassment, and mitigate the burnout of healthcare workers [20,21]. 

Currently, two checklists exist for women workers: the OCHADAI index [17] developed by a national women’s university (i.e., OCHANOMIZU University) in Japan and a checklist developed by the Japan Society for Occupational Health [18]. The former aims to increase the number of women researchers in academia and the later focuses on occupational hazards (e.g., safety procedures). However, both checklists were created without consulting workers; therefore, they do not reflect workers’ needs directly. Additionally, their reliability and validity were not tested. Thus, we hypothesized that developing a checklist for workplaces—to improve working conditions—may encourage women to continue working. Thus, by employing the epidemiological approach, this study aimed to develop a continuous work support checklist for female healthcare workers. 

## 2. Materials and Methods 

### 2.1. Original Items for Scale Development

The concept was first discussed with personnel at the university where this study was conducted. The personnel belonged to the diversity promotion sector; a sector of general affairs and human resources; the nursing department; faculties of medicine, pharma-science, and science technology; and the women’s support center. Original items were created through a series of meetings by referring to previous checklists for a gender-equal society and diversity promotion. Prior to the quantitative survey, a task team consisting of medical doctors, researchers, nurses, and hospital workers and managers in the general affairs division investigated previous literature [17,18] and identified 35 items related to workplace support. To confirm the validity of these 35 items and the wording of each item, 10 researchers of different genders, various age groups, marital statuses, academic positions, and occupations were invited to a pre-test. Subsequently, a final version of 35 items was developed. 

### 2.2. Participants

This survey was conducted at a private university with its main campus located in Tokyo, Japan, and three affiliated hospitals located in Tokyo, Chiba, and Kanagawa. The percentage of women faculty in the university is 24%, and that of nurses is 61% in the hospitals. In February 2017, we recruited 3703 faculty and medical staff working for the three affiliated hospitals, and 977 (women, 65%; medical staff, 80%) returned a self-administered questionnaire (response rate 26%). We excluded missing data on questions related to the scale of workplace support (n = 45) and occupation (n = 25), and faculty whose fields were not related to health or medicine (n = 159). Eventually, 780 (women, 75%) medical professionals became our subjects for analyses. 

This study was approved by the ethics committee in Teikyo University (#TEIRIN 15-141 in 2015). Written informed consent was obtained from all participants.

### 2.3. Questionnaire

The items in the self-administered questionnaire included baseline characteristics (i.e., gender and age), marital status, number of children if any, family member who needs nursing care, occupation, the 35 items related to workplace support for women, and intention to leave. The questions started with the leading sentence, “How important do you think must the workplace consider the following conditions for female workers in health care to continue to work?” Responses for the 35 items were based on a 5-point Likert scale, ranging from 1 for do not agree at all to 5 for agree entirely. The details of the 35 items are presented in Table 1.

To verify if the developed scale functioned to stop women from leaving their workplace, we included a question about the “Intention to leave” the workplace. “Intention to leave” was measured via responses to “I frequently thought about leaving my current workplace”. The response pattern was based on a scale from 1 for not at all to 4 for very much so. Responses 1 and 2, which indicate not at all or no, respectively, were combined, while 3 and 4, indicating somewhat or very much so, respectively, were combined and then grouped into a binary variable. 

### 2.4. Statistical Analysis 

We determined the number of factors based on a scree plot and performed exploratory factor analysis with the maximum likelihood method and promax rotation. Cronbach’s alpha was computed to investigate the reliability of each factor. Each factor was computed based on the summation of the recorded response patterns and divided into binary at its median (i.e., upper half or lower half). We used a chi-square test to understand the characteristics associated with each factor. Finally, to investigate if each factor was associated with “Intention to leave”, a logistic regression model was used to compute odds ratio (OR) along with 95% confidence intervals (CI). If any associations were observed between “Intention to leave” and the factors identified by this study, we suggested that the respondents might not be satisfied with the factors and thus they were more likely to intend to leave their workplace. Stepwise multivariate analyses were performed with forced inclusion of each factor entered one by one. 

All analyses were conducted using SAS (version 9.4, SAS Institute Inc., Cary, NC, USA). The significance level was determined at 0.05 and was two-sided. 

## 3. Results

Table 1 shows a summary of the participants’ individual attributes and the questionnaire survey results. In total, 780 participants were included in this study, of whom 584 (75%) were female, 282 (38%) were in their 20s, 408 (52%) were married, and 438 (56%) were nurses. 

With the initial 35 items, we conducted a maximum likelihood factor analysis using promax rotation, and factor loadings below 0.40 were suppressed. The initial solution based on a scree plot demonstrated four factors with eigenvalues of one or greater. A second factor analysis was performed with loadings below 0.50 to shorten the scale. After excluding items that either cross-loaded or did not load, we extracted four factors with 18 items and computed Cronbach’s alpha for each factor.

The result was a four-factor solution, accounting for 91% of the total variance. Each of the 16 items had moderate to high factor loadings, ranging from 0.62 to 0.95. The first factor contained five items and was labeled “Support for child rearing and home care”. This domain includes a flexible work schedule to support child-rearing and caregivers, availability of a childcare service at the workplace, and flexible work timings. The second factor contained five items and was labeled “Information dissemination”. This domain includes information for women of all ages through social networks, career events, a study group, and booklets. The third factor contained three items and was labeled “Active promotion of women workers to higher positions”. This domain includes active employment and promotion of women. The fourth factor contained three items and was labeled “Consulting and counseling service”. This domain includes consultation and counseling related to careers, harassment, and work–life balance. The details for each factor with 16 items are shown in Table 2. The Cronbach’s alpha values for each factor as well as the overall scale were high, namely 0.90 for Factor 1, 0.90 for Factor 2, 0.92 for Factor 3, and 0.88 for Factor 4, as well as 0.93 for the overall scale, suggesting good reliability and high internal consistency for each factor and the total subscale.

Table 3 shows the characteristics of subjects related to each factor. Each factor was associated with reasonably relevant characteristics. “Support for child rearing and home care” was associated with being female (*p* < 0.001), age (*p* = 0.008), being married (*p* = 0.002), and having a child under seven years old (*p* < 0.001). “Information dissemination” was associated with age (*p* = 0.070), having a child (*p* = 0.065), and faculty (*p* = 0.030). A higher level of “Active promotion of women workers to higher positions” was weakly associated with being female (*p* = 0.073) and providing elderly care (*p* = 0.072). “Consulting and counseling service” was not significantly associated with any characteristics. 

Table 4 indicates factors associated with “Intention to leave” the workplace. Among the four factors, “Support for child rearing and home care” and “Consulting and counseling service” were statistically associated with “Intention to leave” (*p* = 0.002, and *p* = 0.014, respectively). A univariate logistic regression model demonstrated that being female (*p* < 0.001) and being younger (*p* < 0.001) were associated with an increased risk of “Intention to leave”, whereas being married (*p* < 0.001) and having a child (*p* < 0.001) were associated with a decreased risk. Stepwise multivariate models demonstrated that participants who scored higher half points of Factor 1 (OR 1.69, 95% CI: 1.19–2.42) and Factor 4 (OR 1.54, 95% CI: 1.08–2.20) were associated with greater “Intention to leave”. When Factor 1 was included by force, being female (OR 2.14, 95% CI: 1.42–3.22) and being younger (OR 3.19, 95% CI: 1.81–5.64) were associated with an increased risk of “Intention to leave” while being married was associated with a decreased risk (OR 0.59, 95% CI: 0.39–0.90). When Factor 4 was included by force, being female (OR 2.25, 95% CI: 1.50–3.38) and being younger were associated with an increased risk of “Intention to leave” (OR 3.34, 95% CI: 1.89–5.89) while being married was associated with a decreased risk (OR 0.63, 95% CI: 0.42–0.95). 

There were no statistical interactions among sex, age, marriage, and any factors with intention to leave.

## 4. Discussion

### 4.1. Short Summary

In this study, we investigated 780 faculty members of a medical university and health professionals working for the affiliated hospitals in Japan. Prior to the quantitative survey, a task team consisting of medical doctors, researchers, nurses, and hospital workers and managers in the general affairs division identified 35 items related to workplace support. After running exploratory factor analyses with the initial 35 items, we identified four domains with 16 items: “Support for child rearing and home care” (five items), “Information dissemination” (five items), “Active promotion of women workers to higher positions” (three items), and “Consulting and counseling service” (three items). The Cronbach’s alpha values of these four domains ranged from 0.88 to 0.92, suggesting that the scale had high reliability. We found that three of the factors were associated with reasonably relevant characteristics, suggesting that the scale developed in this study was associated with women who are in need. We further found that higher scores in two domains, “Support for child rearing and home care” and “Consulting and counseling service”, were associated with “Intention to leave”, demonstrating that women who intended to leave the workplace underscored the importance of these domains. These results suggest that the scale developed in this study may be helpful for women in healthcare to remain in the workplace.

### 4.2. Interpretation of the Findings

In our study, each factor score was associated with relevant characteristics. These results reflect the current needs of the respondents, supporting our checklist’s validity. Among the four factors, for “Support for child rearing and home care”, participants who were female, in their 30s, married, and had children under seven years old (yet to start compulsory education) were more likely to score higher points, which is reasonably understood because these women are more likely to need this type of support [8,9]. Systems with flexible working hours such as the short-time regular employee system and exemption from night-work in this domain are actually included in Regulations for Child Care and Family Care Leave enforced by the ministry [22]. This indicates that the domain is valid for women to work in balance with their socially prescribed gender roles. The second factor, “Information dissemination and networking”, consists of educational aspects including encouragement for young females to work outside and build their own careers in addition to the information service on child rearing and elderly care. This domain was only associated with faculty members. As our participants were recruited at a private university where faculty were aware of the concept of a gender-equal society, their educational background may explain these associations. The third factor, “Active promotion of women workers to higher positions”, was not associated with any of the characteristics investigated. In Japan, as the Gender Equality Bureau Cabinet Office has released a campaign aimed at “increasing the share of women in leadership positions to at least 30% by 2020” [23], the above factor is in fact quite important; in reality, however, workplaces are still struggling to meet this objective [10,11,13,24]. One reason for the insignificant association with the third factor is that more than half the subjects in our study were nurses, and, as the nursing profession is dominated by women, they automatically receive promotion opportunities. The fourth, “Consulting and counseling service”, was only associated with age. Among the age categories, women in their 50s or older were less likely to score high in this domain compared to other groups. This may be explained by the fact that very few among our study subjects were involved in elderly care, and as their offspring were likely to be grown up, the subjects in their 50s or older were less likely to benefit from the consulting and counseling service.

Logistic regression analysis demonstrated that participants with higher scores for “Support for child-rearing and home care” or “Consulting and counseling service” were associated with an increased risk of “Intention to leave” their workplace. This means that those who highlighted the importance of these two factors for women to work were more likely to intend to leave the workplace, suggesting that these respondents might not be satisfied with these two factors and, accordingly, had intended to leave the workplace. In addition, as mentioned above, women were more likely to score higher for the former factor, indicating that it was significantly important to enable women to work continuously. In 2017, due to the severe shortage of childcare facilities in Japan, 55,433 children were on the national waiting list for places at nursery [25], which makes it impossible for many women to work outside leaving their children alone. According to a Gender Equality Bureau Cabinet Office report in 2018, almost half of women quit their jobs at the time of the birth of their first child [26]. Thus, the association between “Intention to leave” and “Support for child rearing and home care” suggests that, because the participants may not be satisfied with this domain, they were more likely to intend to leave the workplace. Similarly, the observed association between “Consulting and counseling service” and “Intention to leave” may reflect their dissatisfaction in this domain. Our previous study demonstrated that the existence of a consultation and counseling service statistically mitigates burnout among university academics in Japan [12]. Burnout is known to propel workers to quit a job [20,21,27]. Thus, providing consultation and counseling services at the workplace may foster social capital and help workers to remain at their workplace. Additionally, this domain includes “harassment consulting service”. Therefore, those who scored higher for this factor may perceived gender inequality or harassment. This coincides with previous studies [14,15,16]. A previous study in Iran [28] that investigated female health workers demonstrated that trust and social participation in the workplace are beneficial to nurses, patients, and the organization, through improved communication, teamwork, and access to greater information, support, and resources. 

### 4.3. Study Limitations

There are several study limitations to address. First, our sample was recruited from one single medical university and three affiliated hospitals. In addition, because this survey focused on child and nursing care and working environment, our recruited sample might be more likely to be interested in these issues, and be female. Thus, the generalizability of results may be limited to some extent. Second, there are several items thought to be important for women’s work, which were dropped in the process of factor analyses, although the participants placed higher scores on these dropped items. These items included “Substitute workforce for maternity and parental leave”, “Promotion opportunity considering maternity and parental leave”, “No meetings scheduled in the evening or at night”, and “Introduction of a multi-purpose restroom or nursing room”. The potential reason for dropping these may be the insufficient number of items that might have the same domains. The number of initial items might have been relatively few at 35 items, and they may be different depending on the job types and industries. For example, no meetings after 17:00 is now considered a must for female workers to balance between work and family responsibilities. However, in medicine, such a working environment is very difficult to establish considering the characteristics of clinical medicine, and thus our task team initially did not highlight it. Simultaneously, the domains developed in this study may have been biased towards women in clinical health practice. Nevertheless, we invited all related personnel and key persons in related sections in the hospitals and believe that the face validity of the scale developed in this study may be relatively high. In addition, we confirmed high internal consistency and reliability by yielding high Cronbach’s alpha values in all four domains, as well as the overall scores. Third, several other components of reliability (e.g., test–retest reliability) could not be confirmed in this study. The above warrants future studies to examine these areas. Fourth, this study analyzed the relationship between “intention to leave” and factor scores based on the assumption that people who are not satisfied with their current workplace have higher demands regarding the improvement of their workplace. However, participants with higher scores on each item would include those who were satisfied with their current support and wanted it to continue or participants who were interested in those issues regardless of their satisfaction. Thus, the results require careful interpretation. 

### 4.4. Implication

The application of the checklist developed in this study may be useful for both employers and employees in healthcare institutions. For an employer in the fields of health and medicine, the checklist can be used as a tool for work environment development. However, all four domains may be difficult to establish or build if the institution is a small-sized workplace. For example, a small hospital or clinic may not be financially able to provide a consultation section or childcare facility. In that case, instead of a facility or section, alternative efforts should be encouraged, including providing free tickets for nursing, active use of short-time employment, key persons for consultations, or creating a friendly working atmosphere to promote consultation. For employees, periodical assessment by utilizing the checklist may be useful to monitor work satisfaction for each employee, leading to retention of the workforce or decreasing intention to leave a workplace. 

## 5. Conclusions

Healthcare work is very popular among women, but, In Japan, women readily quit a job because of gender role responsibilities. Using 780 faculty members and healthcare professionals from one medical university in Japan, we developed a scale for continuous work support for female healthcare workers. Our analysis identified four factors with 16 items, labeled “Support for child rearing and home care”, “Information dissemination”, “Active promotion of women workers to higher positions”, and “Consulting and counseling service”. The present study confirmed that these domains generally reflect the current needs of female healthcare workers. Using the checklist to improve the work environment may help prevent women from quitting work and instead continue at their workplace.

## Figures and Tables

**Table 1:** Characteristics of our subjects.

		Total		Female (n = 584)		Male (n = 196)	
		n	%	n	%	n	%
Age group							
	21–29	282	37.5	242	44.6	40	19.0
	30–39	164	21.8	127	23.4	37	17.6
	40–49	170	22.6	119	22.0	51	24.3
	50–70	136	18.1	54	10.0	82	39.1
Marital status							
	Single *	371	47.6	318	56.4	53	24.8
	Married	408	52.4	246	43.6	161	75.2
Presence of child							
	None	409	55.2	337	63.2	72	34.8
	1–	332	44.8	196	36.8	135	65.2
Elderly care							
	Not involved	716	92.1	516	92.0	199	92.6
	Yes	61	7.9	45	8.0	16	7.4
Occupation							
	Doctor	154	19.7	37	6.5	116	54.0
	Pharmacist	50	6.4	32	5.7	18	8.4
	Nurse	438	56.2	410	72.7	28	13.0
	Technician	138	17.7	85	15.1	53	24.6
Intention to leave							
	No	341	44.3	201	36.2	139	65.6
	Yes	428	55.7	355	63.9	73	34.4
							
		**mean**	**sd**	**mean**	**sd**	**mean**	**sd**
The workplace must meet the following conditions….							
1)	An organization to support women	4.10	0.89	4.09	0.86	4.11	0.98
2)	An external evaluation committee for women’s support	3.92	0.88	3.94	0.84	3.87	0.99
3)	A highly fair and transparent hiring and promotion system	4.12	0.84	4.11	0.81	4.13	0.92
4)	Active employment of women	3.77	0.91	3.81	0.87	3.67	1.02
5)	Promotion of women to management positions	3.71	0.90	3.74	0.87	3.62	0.97
6)	Active employment of women by focusing on job category or classification	3.59	0.91	3.66	0.84	3.39	1.03
7)	Investigation of the ratio of women by field and job position	3.79	0.88	3.80	0.83	3.76	1.01
8)	No meetings scheduled in the evening or at night	4.19	0.96	4.30	0.89	3.91	1.06
9)	Encouraging men to take parental leave	4.02	0.92	4.05	0.89	3.95	0.98
10)	Investigation of actual working hours by sex	3.57	0.99	3.58	0.99	3.52	0.99
11)	Investigation of the actual tenure separated by sex	3.58	0.97	3.59	0.97	3.56	0.99
12)	Investigation into the work-life balance	4.12	0.86	4.16	0.83	4.00	0.95
13)	A room for women to rest	3.66	1.03	3.70	0.99	3.56	1.12
14)	A multi-purpose restroom or nursing room	4.18	0.83	4.18	0.82	4.20	0.86
15)	A playroom for kids	4.01	0.89	4.02	0.89	3.97	0.90
16)	A childcare facility	4.54	0.72	4.55	0.70	4.52	0.79
17)	A daycare for sick and recovering children	4.49	0.75	4.50	0.74	4.45	0.77
18)	A flexible work schedule to support child-rearing	4.46	0.75	4.51	0.72	4.34	0.83
19)	A flexible work schedule to support caregivers	4.45	0.74	4.51	0.70	4.29	0.83
20)	Exemption from late-night shifts during pregnancy and child-rearing period	4.48	0.75	4.53	0.72	4.34	0.81
21)	Performance evaluation that is not disadvantageous to career advancement	4.31	0.80	4.34	0.78	4.23	0.84
22)	Harassment counseling service	4.17	0.85	4.19	0.84	4.14	0.89
23)	Career consultation service	4.01	0.86	4.04	0.85	3.96	0.90
24)	Work-life balance consultation service	4.07	0.86	4.10	0.85	3.98	0.90
25)	Substitute staff for maternity leave	4.30	0.84	4.33	0.83	4.25	0.84
26)	A mentor to support women	3.79	0.83	3.81	0.78	3.74	0.94
27)	Role models in the workplace	4.03	0.88	4.06	0.84	3.95	0.96
28)	A booklet for childcare and nursing care support	3.56	0.94	3.59	0.93	3.48	0.97
29)	A network among women	3.74	0.86	3.74	0.84	3.72	0.92
30)	Career events for junior and senior high school girls	3.42	0.91	3.48	0.88	3.29	0.99
31)	Information to support women for all officials (homepage, mail, newspaper, bulletin, etc.)	3.67	0.91	3.71	0.87	3.58	0.98
32)	A study group and exchange meeting about parenting and nursing care support	3.70	0.85	3.71	0.81	3.66	0.96
33)	Raising awareness among men about support for child-rearing and nursing care	4.02	0.84	4.04	0.82	3.96	0.89
34)	Raising management awareness about support for child-rearing or caregivers	4.16	0.81	4.17	0.80	4.13	0.86
35)	Raising awareness about understanding of diversity (gender, disability, cultural differences, etc.)	4.04	0.84	4.04	0.81	4.04	0.92
Total numbers that do not reach 780 indicates missing in the category level.							
* Single includes divorced and widowed.							

**Table 2:** Exploratory factor analysis result.

		Factor 1	Factor 2	Factor 3	Factor 4
**Factor 1 “Support for child rearing and home care”** α=0.90					
	A flexible work schedule to support child rearing	0.947	0.041	–0.028	–0.022
	A flexible work schedule to support caregivers	0.846	–0.041	0.018	0.089
	A child care facility	0.742	0.045	–0.001	–0.047
	A day care for sick and recovering children	0.729	0.012	0.006	–0.013
	Exemption from late-night shifts during pregnancy and child-rearing period.	0.622	–0.018	0.063	0.136
**Factor 2 “Information dissemination”** α = 0.90					
	A network among women	0.083	0.779	0.004	–0.043
	Career events for junior and senior high school girls	–0.072	0.776	0.061	0.027
	A study group and exchange meeting about parenting and nursing care support	0.063	0.770	–0.037	0.051
	Information to support women for all officials (homepage, mail, newspaper, bulletin, etc.)	0.051	0.751	0.065	0.039
	A booklet for childcare and nursing care support	–0.047	0.751	0.028	0.040
**Factor 3 “Active promotion of women workers to higher positions”** α=0.92					
	Promotion of women to management positions	0.023	–0.009	0.920	–0.014
	Active employment of women	0.035	0.044	0.828	–0.003
	Active employment of women by focusing on job category or classification	–0.021	0.099	0.672	0.062
**Factor 4 “Consulting and counseling service”** α=0.88					
	Career consultation service	–0.020	0.071	0.020	0.902
	Harassment counseling service	0.148	0.009	0.001	0.765
	Work-life balance consultation service	0.066	0.087	0.028	0.756
Factor correlation					
Factor1		1.00			
Factor2		0.45	1.00		
Factor3		0.42	0.56	1.00	
Factor4		0.58	0.60	0.52	1.00

**Table 3:** Characteristics of subjects related to each factor.

		Factor 1 “Support for Child Rearing and Home Care”			Factor 2 “Information Dissemination”			Factor 3 “Active Promotion of Women Workers to Higher Positions”			Factor 4 “Consulting and Counseling Service”		
		Upper Half	Lower Half	*p*	Upper Half	Lower Half	*p*	Upper Half	Lower Half	*p*	Upper Half	Lower Half	*p*
		n (%)	n (%)		n (%)	n (%)		n (%)	n (%)		n (%)	n (%)	
Sex				<0.001			0.528			0.073			0.211
	Male	82(38)	131(62)		101(48)	110(52)		89(42)	124(58)		82(38)	131(62)	
	Female	294(53)	264(47)		252(45)	304(55)		272(49)	283(51)		243(43)	316(57)	
Age				0.008			0.070			0.503			0.041
	21–29	118(43)	159(57)		120(44)	155(56)		128(47)	147(53)		100(36)	178(64)	
	30–39	97(59)	67(41)		69(43)	93(57)		71(44)	92(56)		74(45)	89(55)	
	40–49	85(50)	85(50)		83(49)	87(51)		82(48)	88(52)		83(49)	87(51)	
	50–70	61(46)	73(54)		75(56)	59(44)		70(52)	64(48)		55(41)	80(59)	
Marital status				0.002			0.305			0.836			0.609
	Single	157(43)	210(57)		160(44)	203(56)		173(48)	191(52)		151(41)	216(59)	
	Married	219(54)	185(46)		193(48)	211(52)		189(47)	215(53)		174(43)	231(57)	
Children				0.066			0.065			0.461			0.916
	None	185(46)	218(54)		169(42)	231(58)		183(46)	217(54)		170(42)	234(58)	
	1–	174(53)	156(47)		162(49)	168(51)		160(48)	170(52)		138(42)	193(58)	
Children under 7 years old				<0.001			0.558			0.331			0.467
	None	268(46)	321(55)		263(45)	323(55)		281(48)	305(52)		252(43)	339(57)	
	1–	92(63)	53(37)		69(48)	76(52)		63(43)	82(57)		57(39)	88(61)	
Elderly care				0.199			0.143			0.072			0.065
	Not involved	341(48)	369(52)		319(45)	387(55)		327(46)	380(54)		292(41)	419(59)	
	Yes	34(57)	26(43)		33(55)	27(45)		35(58)	25(42)		32(53)	28(47)	
Faculty				0.429			0.030			0.471			0.817
	No	229(51)	219(49)		202(45)	244(55)		221(49)	227(51)		192(43)	257(57)	
	Yes	96(48)	105(52)		109(55)	91(46)		93(46)	108(54)		84(42)	117(58)	

**Table 4:** The influence of four factors on intention to leave workplace.

		Intention to Leave			Logistic Regression Analyses					
							Stepwise Multivariate Model			
		(+)	(–)		Crude		Factor 1 “Support for Child Rearing and Home Care”		Factor 4 “Consulting and Counseling Service”	
		n (%)	n (%)	*p*	OR	95%CI	OR	95%CI	OR	95%CI
**Factor 1**				0.002	1.59	1.19–2.12	1.69	1.19–2.42	–	–
	Upper half	227(62)	142(38)							
	Lower half	198(50)	197(50)							
**Factor 2**				0.589	0.92	0.69–1.23	–	–	–	–
	Upper half	190(55)	158(45)							
	Lower half	233(57)	179(43)							
**Factor 3**				0.211	1.20	0.90–1.60	–	–	–	–
	Upper half	207(58)	150(42)							
	Lower half	216(53)	188(47)							
**Factor 4**				0.014	1.44	1.08–1.93	–	–	1.54	1.08–2.20
	Upper half	196(61)	126(39)							
	Lower half	230(52)	213(48)							
**Sex**										
	Male	73(34)	139(66)	<0.001	1.00		1.00	–	1.00	–
	Female	355(64)	201(36)		3.36	2.41–4.69	2.14	1.42–3.22	2.25	1.50–3.38
**Age**				<0.001						
	50–70	44(32)	92(68)		1.00		1.00	–	1.00	–
	21–29	197(71)	81(29)		5.09	3.27–7.92	3.19	1.81–5.64	3.34	1.89–5.89
	30–39	93(57)	69(43)		2.82	1.75–4.53	1.80	1.80–1.05	1.91	1.12–3.26
	40–49	76(46)	90(54)		1.77	1.1–2.83	1.39	0.82–2.33	1.35	0.80–2.27
**Marital status**				<0.001						
	Single	246(67)	122(33)		1.00		1.00	–	1.00	–
	Married	182(46)	218(55)		0.41	0.31–0.56	0.59	0.39–0.90	0.63	0.42–0.95
**Children**				<0.001			–	–	–	–
	None	263(65)	139(35)		1.00					
	1–	140(43)	188(57)		0.39	0.29–0.53				
**Children under 7 years old**				0.450			–	–	–	–
	None	329(56)	259(44)		1.00					
	1–	75(52)	68(48)		0.87	0.60–1.25				
**Elderly care**				0.813			–	–	–	–
	Not involved	393(56)	313(44)		1.00					
	Yes	33(54)	28(46)		0.94	0.56–1.59				
**Faculty**				<0.001			–	–	–	–
	No	284(64)	162(36)		1.00					
	Yes	67(34)	133(67)		0.29	0.20–0.41				
